# *Valerianapraecipitis* (Caprifoliaceae), a species new to science and endemic to Central Chile

**DOI:** 10.3897/phytokeys.189.73959

**Published:** 2022-02-04

**Authors:** Alejandro E. Villarroel, Kora Menegoz, Carlos Le Quesne, Ricardo Moreno-Gonzalez

**Affiliations:** 1 Departamento de Biología, Facultad de Ciencias, Universidad de La Serena, Av. Raúl Bitrán Nachary 1305, La Serena, Chile; 2 Instituto de Conservación, Biodiversidad y Territorio, Facultad de Ciencias Forestales y Recursos Naturales, Universidad Austral de Chile, Independencia 641, Valdivia, Chile; 3 Independent researcher. Macal alto S/N, San Fabián de Alico, Chile; 4 Laboratorio de Dendrocronología y Cambio Global, Instituto de Conservación, Biodiversidad y Territorio, Universidad Austral de Chile, Valdivia 5110566, Chile; 5 Department of Palynology and Climate Dynamics, University of Göttingen, Wilhelm-Weber-Str. 2a, 37073 Göttingen, Germany; 6 Laboratorio de Biodiversidad y Ecología del Dosel, Instituto de Conservación, Biodiversidad y Territorio, Universidad Austral de Chile, Independencia 641, Valdivia, Chile

**Keywords:** Andes, biodiversity, cliffs flora, Ñuble Region, taxonomy

## Abstract

The species *Valerianapraecipitis* (Caprifoliaceae), new to science and endemic to the Ñuble Region, Central Chile, is formally described. Morphological data support its placement in a new species, clearly different from *V.philippiana*. A detailed description, insights about its habitat and ecology, distribution map and illustration are provided. A table of comparison is also given with the morphological characters discriminating *V.praecipitis* from *V.philippiana*. The species is assessed as Endangered (EN) under the IUCN categories.

## ﻿Introduction

﻿The Valerianaceae family was included in the Caprifoliaceae family by the Angiosperm Phylogeny Group III (APG III 2009). Although some authors continued using Valerianaceae for several years (e.g., Kutschker 2011), the family remained within the Caprifoliaceae in the APG IV (2016). Caprifoliaceae includes about 960 species in approximately 41 genera (Wang et al. 2020), having an almost cosmopolitan distribution, with centres of diversity in eastern North America and eastern Asia (Xu and Chang 2017).

*Valeriana* is one of the most diverse genera within the Caprifoliaceae, with a worldwide distribution, and centres of diversity in tropical areas of Central-America and to the south along the Andes mountains (Kutschker and Morrone 2012). In South-America, *Valeriana* species are present in Peru, Bolivia, Brazil, Chile and Argentina (Zuloaga et al. 2008). In Chile, Borsini (1966) described 42 species, while the most recent assessment identified 40 species in the Andes mountains of Chile and Argentina, between latitudes 33° and 56° S (Kutschker 2011). This area was identified as a centre of diversification of *Valeriana* species (Kutschker and Morrone 2012). In Chile 44 species are accepted, of which 18 are endemic, and interestingly, to date no introduced species has been found so far (Rodríguez et al. 2018). All species are herbaceous (Rodríguez et al. 2018) and many of them are distributed in high-altitude mountainous locations, usually above 1000 m elevation (e.g., *V.stricta* Clos, *V.philippiana* Briq.). They can also be restricted to dry soils (e.g., *V.corynodes* Borsini) or in permanently wet soils (e.g., *V.fonckii* Phil.). Recently, only one species new to science has been formally described, *V.nahuelbutae* Penneck. (Penneckamp 2020) and few new geographical locations have been found for other species (e.g., Kutschker 2011; Teillier et al. 2020). Furthermore, for most species little is known about their ecology or ethnobotany, and to this day, only one of the Chilean species have had formal conservation assessments completed according to the IUCN criteria (*Valerianasenecioides* Phil.; MMA 2021).

**Figure 1.**
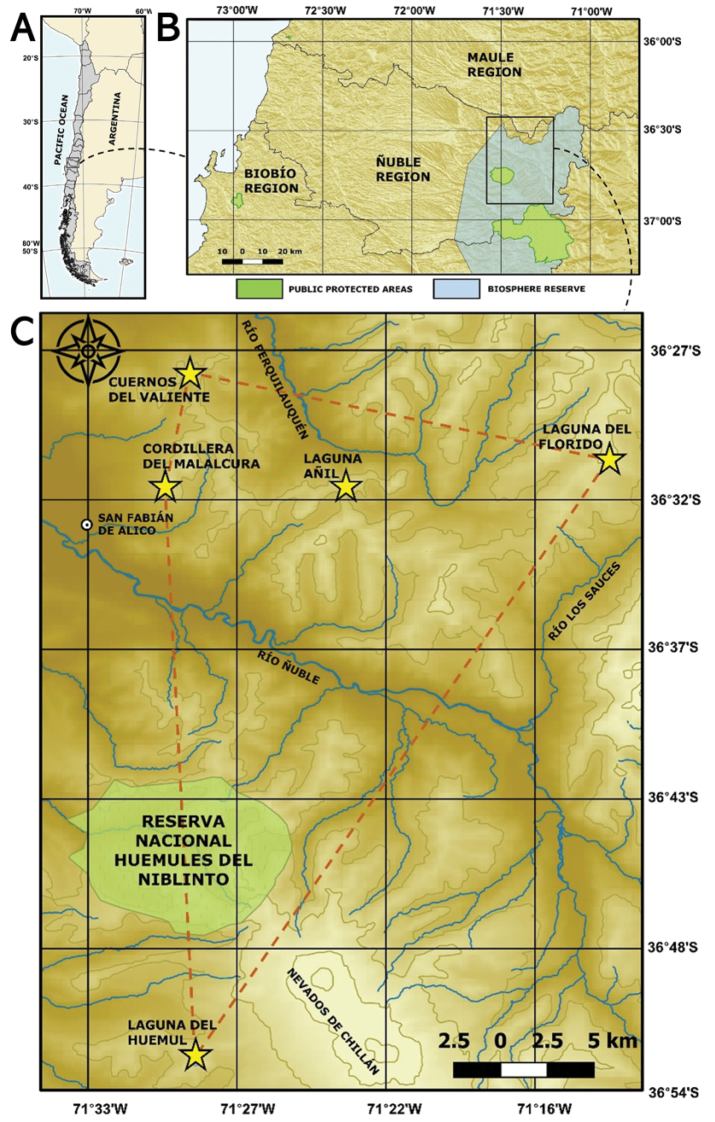
Distribution maps of *Valerianapraecipitis***A** Chile **B** Ñuble Region **C** sites of *V.praecipitis* (yellow stars) and extent of occurrence (red line).

The Central Chilean Andes mountains are recognised as a centre for endemism in South-America (e.g., Villagrán and Hinojosa 1997; Arroyo et al. 2006). In addition, the Andean ranges of the Ñuble Region were declared as a Biosphere Reserve in 2011 (San Martín 2014; Fig. 1) and this area is of increasing interest since a new genus was recently described (Villarroel et al. 2021). However, several species in the region remain severely threatened (Hechenleitner et al. 2005). Intensive land-use changes due to human activities have affected vast extensions of almost all types of vegetation in Central Chile, including high-Andean habitats (Arroyo et al. 2006). In addition, global climate change is a serious threat to montane habitats that have reduced in size, are geographically isolated, and where the environmental conditions have changed significantly across small distances due to steep slopes (Báez et al. 2016).

In this manuscript we describe a species of *Valeriana* located in the Central Chilean Andes at around 36° S latitude and clearly distinguishable from other species by its remarkable silvery-green basal leaves. In the following sections, a detailed account on how the species was discovered is given as a formal description. In addition, a distribution map, insights about its habitat and ecology, conservation status, and illustrations are provided.

## Methods

### Herbarium and Fieldwork

During the austral spring-summer between 2015–2021 several botanical explorations were carried out in the Andean ranges within the locality of San Fabián de Alico, Punilla Province, Ñuble Region, Chile (Fig. 1), by means of rock-climbing in the Cordillera del Malalcura and Cuernos del Valiente and treks to Laguna del Florido. In addition, seven expeditions were made to Laguna Añil which fell within the scope of our research project entitled “Richness and distribution of the flora along an altitudinal gradient in Bullileo creek, Ñuble Region” (Villarroel 2019). During these excursions, a species of *Valeriana* that could not be assigned to any known species for Chile and Argentina was found. Its habitat and characters such as plant size and root system were observed and measured in the field.

Herbarium specimens were collected from Laguna Añil, and distributed to the herbaria of CONC, EIF, JBN and SGO (acronyms after Thiers 2016). As our preliminary observations of the plant and the key to *Valeriana* genus (Kutschker 2011) suggested similarities with *Valerianaphilippiana* Briq., (e.g., basal leaves silvery-green colour and pinnatisect, terminal lobe size equal to bigger, fleshy texture, arranged in an imbricate rosette, fruits pubescent, pappus plumose), we examined the herbarium specimen at SGO to determine the morphological differences between both species. Also, online digital images of specimens available on CONC, E and US herbaria websites were studied, as well as literature (Borsini 1966; Kutschker 2008, 2011). The taxonomic treatment was prepared after examining all available specimens.

### Morphological analysis

The morphological study was based on observations and measurement of fresh and dried specimens. Detailed photographs of fresh material were taken in the field to document the overall plant morphology and especially the floral structure. In order to accurately describe the vegetative and reproductive morphology of the collected plants, dry and rehydrated specimens were dissected. Ovary, fruits, flowers and leaf details were photographed with a zoom lens and subsequently observed under a binocular microscope. Terminology for describing *Valeriana* plant parts followed Borsini (1966) and Kutschker (2008, 2011).

### Conservation status

The conservation status assessment of the species used the International Union for Conservation of Nature criteria (IUCN Standards and Petitions Committee 2019). The extent of occurrence (EOO) and area of occupancy (AOO) were calculated using GeoCat (Bachman et al. 2011).

## Taxonomic treatment

### Valeriana praecipitis

Taxon classificationPlantaeDipsacalesCaprifoliaceae

A.E. Villarroel & Menegoz, sp. nov.

36E06224-88C0-54A5-8136-0787610D4E49

urn:lsid:ipni.org:names:77254613-1

Figures 2 , 3 , 4

#### Type.

**Figure 2.**
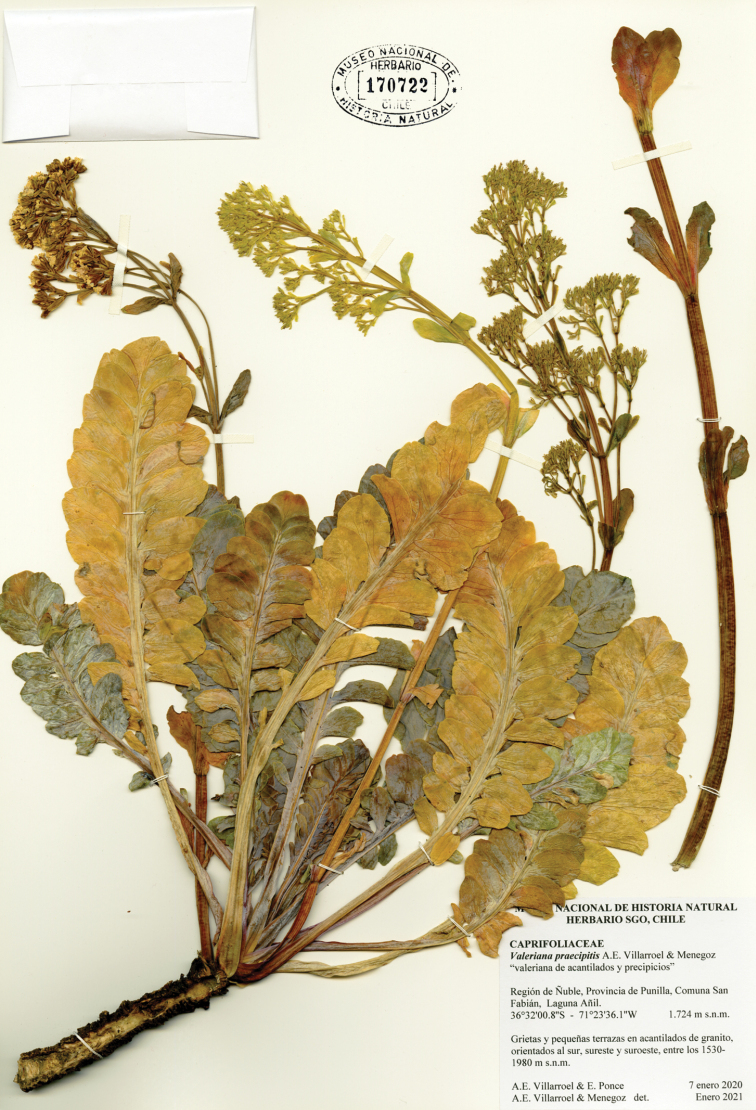
Holotype of *Valerianapraecipitis*.

Chile. Ñuble Region, Punilla Province, San Fabián de Alico, Laguna Añil, crevices and small terraces of granite cliffs, 1724 m elevation, 36°32'00.8"S, 71°23'36.1"W, 7 January 2020, *A.E. Villarroel* & *E. Ponce s.n.*, (holotype SGO!); 1724 m elevation, 36°32'00.8"S, 71°23'36.1"W, 3 February 2021, *A.E. Villarroel* & *R. Neira* (paratypes EIF!, JBN!); 1650 m elevation, 36°32'2.28"S, 71°23'26.62"W, 7 December 2020, *K. Menegoz & G. Ossa* (paratypes CONC!) (Fig. 2). Laguna Añil is the only site where herbarium specimens were collected.

#### Diagnosis.

The habit and macro-morphology of *Valerianapraecipitis* is similar to *Valerianaphilippiana*, but differs by its height (including flower stem) that can reach 65.5 cm (vs. 20 cm), rhizome woody, reaching more than 30 cm long and up to 20 mm diameter (vs. semi-woody, to 14 cm long, to 8 mm diam.), basal leaves pinnatisect to pinnatipartite, up to 26 cm long (vs. pinnatilobed to pinnatisect, to 8 cm long), petiole glabrous (vs. pubescent), lobes 1–35 mm long, 1–24 mm wide (vs. 4–8 × 3–7 mm), upper leaves oblanceolate, 14–40 mm long, 5–19 mm wide, margin entire to irregularly undulate or sinuate, (vs. oblong, 6–10 × 3–5 mm, entire), bracts oblanceolate to oblong, up to 20 mm long (vs. oblong, to 7 mm long), bracteoles spathulate to oblong, 3–7 mm long, entire (vs. oblong, 2.5–4.5 mm, erose), inflorescence a relatively diffuse thyrse or compound dichasial cyme (vs. dense compound dichasial cyme, contracted), corolla up to 4.5 mm long (vs. up to 4 mm), stamens 3 mm long (vs. 2 mm), stigma 0.2 mm long (vs. 0.5 mm), growing on cliffs that remain humid all-year (vs. well-drained rocky soils), and endemic to the Ñuble Region (vs. in Chile, *V.philippiana* can be found in Los Lagos, Aysén and Magallanes Regions) (Table 1).

**Table 1.** Morphological differences between *Valerianapraecipitis* and *V.philippiana*. Based on Borsini (1966) and Kutschker (2008, 2011). In bold: character unique to *V.praecipitis*.

***Species***	***V.praecipitis***	***V.philippiana***
Habit	Perennial herb, hemicryptophyte, simple or branched	Perennial herb, hemicryptophyte, simple or branched
Height with inflorescence	**26–65.5 cm**	To 20 cm
Taproot rhizome	Circular, **reaching more than 30 cm long**, **8–20 mm diameter**, sometimes stoloniferous, **woody**	Circular, reaching 14 cm long, 5–8 mm diameter, sometimes stoloniferous, semi-woody
Stem	3.5–6 mm diameter, with very short internodes, forming a basal rosette	3–6 mm diameter, with very short internodes, forming a basal rosette
Basal leaves	Pinnatisect **to pinnatipartite**, oblong, **up to 26 cm long**	Pinnatilobed to pinnatisect, oblong, up to 8 cm long
Lobes	Orbicular to obovate, base attenuate, **1–35 × 1–24 mm**, overlapped, glabrous, fleshy. **In the field, leaf lobes are expanded**	Orbicular to obovate, base attenuate, 4–8 × 3–7 mm, overlapped, glabrous, fleshy. In the field, leaf lobes are quite folded
Petioles	Canaliculated, **glabrous**	Canaliculated, pubescent
Upper leaves	**Oblanceolate, 14–40 × 5–19 mm**, margin entire **to irregularly undulate or sinuate**	Oblong, 6–10 × 3–5 mm, margin entire
Bracts	**Oblanceolate** to oblong, **up to 20 mm long**	Oblong, up to 7 mm long
Bracteoles	**Spatulate** to oblong, **3–7 mm long, margin entire**	Oblong, 2.5–4.5 mm long, margin erose
Inflorescence	**A relatively diffuse thyrse** or compound dichasial cyme	Dense compound dichasial cyme
Flowers	Hermaphrodite; corolla infundibuliform; **corolla tube 3.5–4.5** mm long, base slightly gibbous; corolla lobes oblong to obovate, 1–1.5 × 1–1.7 mm; **stamens 3 mm long**, exerted; ovary incipient sterile locules; **style 2.2 mm long**; stigma lobed laminate to lamellate, **less than 0.2 mm**	Hermaphrodite; corolla infundibuliform-campanulate; corolla tube 4 mm long, base gibbous; corolla lobes oblong to obovate, 1.5 × 1.5–2 mm; stamens 2 mm long, exerted; ovary incipient sterile locules; style 2.5 mm long; stigma lobed lamellate, 0.5 mm
Fruits	Ellipsoid, **3 × 1 mm**, pubescent; pappus plumose, bristles 11, **3.5 mm long**	Ellipsoid, 3–4 × 2 mm, pubescent; pappus plumose, bristles 11–13, 5–7 mm long

#### Description.

Perennial ***herb***, hemicryptophyte, erect or lax when cliff-hanging, simple or branched from the upper part of the taproot, 4–25 cm tall (26–65.5 cm with inflorescence), 4–28.5 cm wide. ***Rhizome*** is dark brown, thick, circular, simple, sometimes branched, reaching more than 30 cm long, 8–20 mm diameter, vertical to lateral, sometimes stoloniferous, woody, tortuous, rough, fetid. ***Secondary*-*tertiary roots***, numerous, located in the first 3 cm of the upper part of the taproot. ***Stem*** merging into the taproot, 3.5–6 mm diameter, with short internodes, forming a basal rosette with 9–25 whorled leaves. ***Basal leaves*** deciduous, silvery-green turning yellow-brown at the end of summer, simple, petiolate, pinnatisect, sometimes becoming gradually pinnatipartite at the apex (mainly young leaves), oblong, generally symmetric; ***blade*** 3–16 cm long (4–26 cm with petiole), 1.5–6.3 cm wide, glabrous, fleshy, with reticulated veins; ***petiole*** green turning purple towards the base, canaliculated, up to 14.5 cm long, 3–13 mm wide at the base, 2–7 mm wide at the blade base, entire, glabrous, midrib visible; ***lateral lobes*** opposite to subopposite, superimposed, orbicular to obovate, base attenuate, apex rounded to retuse, margin entire to slightly undulate and involute, 6–26 per blade; larger lobes located in the centre of the blade, 9–35 × 7–22 mm; smaller lobes located at the base of the blade, 1–15 × 1–8 mm; ***terminal lobe*** orbicular to obovate, 6.5–23.5 × 6–24 mm, base attenuate, apex rounded to obtuse, occasionally retuse, margin entire to irregularly undulate or lobed. ***Inflorescence*** a relatively diffuse thyrse or terminal compound dichasium, sometimes corymboid. ***Floral stem*** purple at the base, light green towards the flowers, erect, circular, 28.6–60 cm long, 3.5–6 mm diameter at the base, gradually thinner towards the flowers (1.5–2.9 mm), striated, 5–9 internodes (their length decreasing from base toward the apex), branched in the upper half (2–27 cm long) with 1–6 lateral ascending branch pairs (forming partial inflorescences). ***Upper leaves*** green, simple, sessile, oblanceolate, 14–40 × 5–19 mm, opposite, decussate, arranged every 2.7–13.5 cm on the flower stem, leaves’ size decreasing from base toward the inflorescence, margin entire to irregularly undulate or sinuate, base decurrent, apex acute to rounded, occasionally retuse, glabrous, less fleshy than basal leaves, reticulated veins. ***Bracts*** green, simple, sessile, oblanceolate to oblong, 7–21.2 × 1–7 mm, decreasing in size towards the inflorescence, opposite, decussate, margin entire, base decurrent, apex variable (acute, rounded or retuse), glabrous, less fleshy than upper leaves, reticulated veins. ***Bracteoles*** green, sometimes turning purple towards the apex, simple, sessile, spathulate to oblong, 3–7.5 × 0.5–2 mm, decreasing in size towards the inflorescence, opposite, decussate, margin entire, base decurrent, apex rounded to retuse, glabrous, less fleshy than upper leaves. ***Flowers*** hermaphrodite, pentamerous, sessile; ***calyx*** green and purple at the top, inconspicuous, fused segments forming a wavy ring, 0.3 mm, pubescent, adnate to the infer ovary, accrescent and persistent on fruit modified into feathery structures forming the pappus; ***corolla*** 5, fused petals, white, although buds sometimes with purple-pink tinges, infundibuliform, glabrous, 4–4.5 mm wide; ***corolla tube*** 3.5–4.5 mm long, base slightly gibbous; ***corolla lobes*** oblong to obovate, 1–1.5 × 1–1.7 mm, perpendicular or slightly inclined in relation to the corolla tube; ***stamens*** 3, white, filiform, 3 mm long, exerted, attached in the lower third of the tube; ***anthers*** light yellow, ellipsoid, bithecal, dorsifixed, deciduous; ***ovary*** inferior, green, tricarpellate, trilocular with 1 fertile locule and 2 incipient sterile locules; ***style*** 1, white, filiform, 2.2 mm long; ***stigma*** trifid, lobed, laminate to lamellate, less than 0.2 mm. ***Fruit*** an achene, yellow-green at the base, turning purple towards the apex, ellipsoid, triquetrous, 3 × 1 mm, pubescent, longitudinally striated on one face, calyx persistent, ***pappus*** plumose, 0.5 mm diameter at base, bristles 11, purple-reddish colour, 3.5 mm long, with hairs 0.5 mm long.

**Figure 3.**
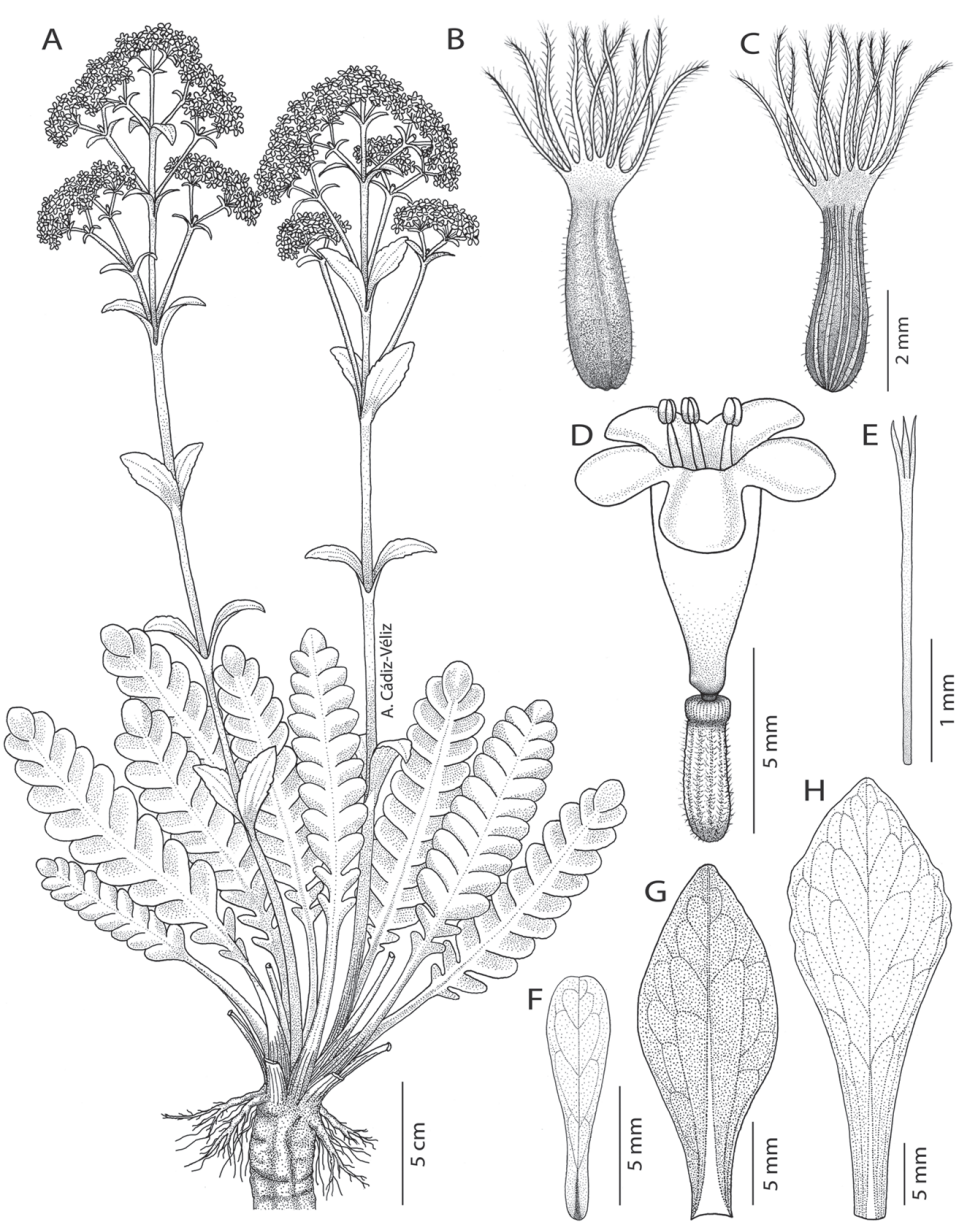
*Valerianapraecipitis***A** habit **B, C** fruit **D** detail of flower **E** stigma **F** bracteole **G** bract **H** upper leave. Drawn by Arón Cádiz-Véliz.

**Figure 4.**
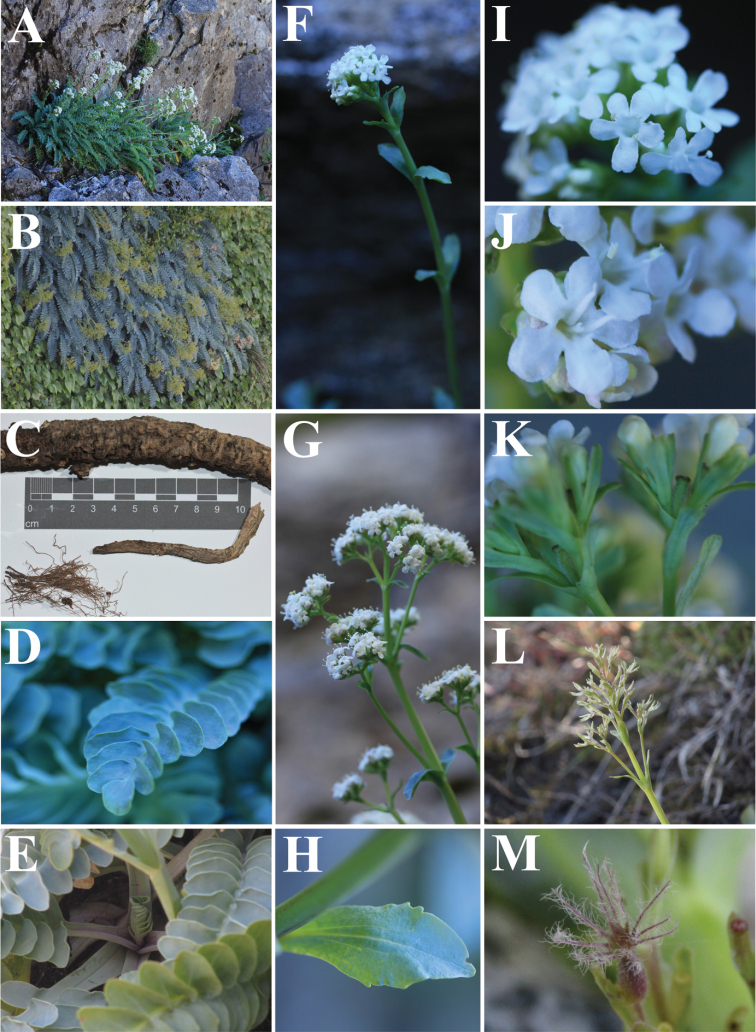
*Valerianapraecipitis***A, B** plants growing in natural habitat **C** rhizome, secondary-tertiary roots **D** basal leaves with lobes detail **E** petioles **F** floral stem, corymboid inflorescence **G** floral stem, thyrse inflorescence **H** upper leaves **I, J** detail of flowers (stamens, style) **K** bracteoles, ovary, calyx **L** dry inflorescence, bracts, bracteoles **M** fruit, pappus. Photographed by Alejandro E. Villarroel and Kora Menegoz.

#### Etymology.

The specific epithet refers to cliff faces inhabited by these plants. The name means “*Valeriana* of cliffs” (latin *praeceps* = steep place, precipice, dangerous; genitive *praecipitis*).

#### Phenology.

Flowering from November to December; fruiting from January to February.

#### Distribution and habitat.

**Figure 5.**
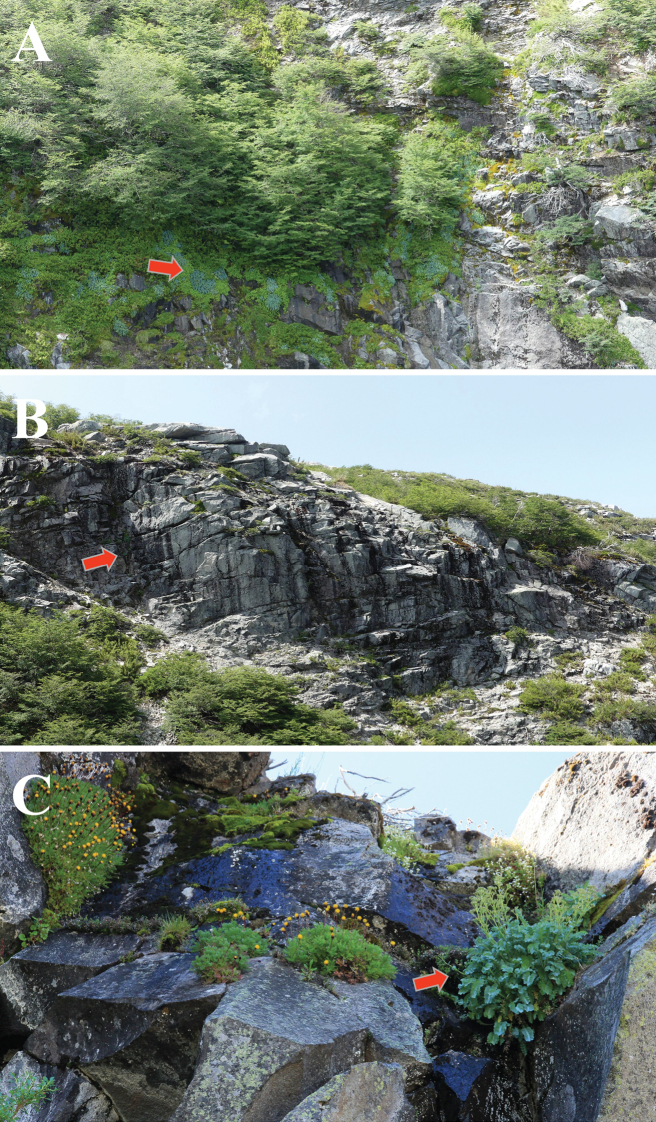
Habitat of *Valerianapraecipitis* (red arrow indicates the species) **A, B** Laguna Añil **C** Laguna del Florido. Photographed by Alejandro E. Villarroel and Kora Menegoz.

Endemic to the Andean ranges of the Ñuble Region, Chile (Fig. 1A, B). Specifically, 5 sites are known so far (Fig. 1C): Laguna Añil (1724 m elevation, 36°32'00.8"S, 71°23'36.1"W; 1650 m elevation, 36°32'2.28"S, 71°23'26.62"W); Cuernos del Valiente (1530 m elevation, 36°27'46.31"S, 71°29'14.41"W), Cordillera del Malalcura (1700 m elevation, 36°32'0.23"S, 71°30'7.59"W), Laguna del Florido (1980 m elevation, 36°30'55.07"S, 71°14'1.20"W), and the last at Laguna del Huemul (1970 m elevation, 36°52'40.11"S, 71°29'6.33"W, this locality was found by Eitel Thielemann). The maximum distance between the sites is 38 km. The species usually grows at high elevations (1530–1980 m), in crevices and small terraces of south, southeast and southwest facing cliffs (Fig. 5). Due to snow melting and low sun exposure, these sites remain humid during the dry season. Two closest weather stations, Punilla (840 m elevation) and Caracol (725 m elevation), indicate that the average annual temperature is 11.8 °C and the average total annual precipitation 2150 mm for the period 1965–2012 (DGA 2018). Although at a lower elevation than *V.praecipitis* altitudinal range, a private pluviometer in the village of San Fabián de Alico (447 m elevation) recorded an annual average precipitation of 1399 mm for the period 2017–2020 (Covarrubias J.C., pers. comm.). In addition, to get a better impression of the local conditions during year 2020 temperature and humidity data loggers HOBO U23 Pro v2 (Onset Computer Corporation, Massachusetts, USA) registered an average annual temperature of 6.7 °C, with a minimum of -2.8 °C, a maximum of 24.1 °C, and an average humidity 60.12% at Laguna Añil; and an average annual temperature of 8.7 °C, with a minimum of -4.5 °C, a maximum of 24.9 °C, and an average humidity of 66.39 % in Cordillera del Malalcura.

#### Associated vegetation.

From a phytogeographical point of view, *Valerianapraecipitis* is part of two vegetational formations and three vegetation belts (Luebert and Pliscoff 2017). The vegetation formation at Laguna Añil, Cuernos del Valiente and Cordillera del Malalcura corresponds to Deciduous forest, and the vegetation belt to the Andean temperate deciduous forest of *Nothofaguspumilio* (Poeppig & Endlicher) Krasser (Nothofagaceae) and *Azaraalpina* Poeppig & Endlicher (Salicaceae) (Fig. 5A, B); the vegetation formation in Laguna del Florido corresponds to the Altitude low scrub, and the vegetation belt to the Andean Mediterranean low scrub of *Laretiaacaulis* (Cav.) Gillies & Hook. (Apiaceae) and *Berberisempetrifolia* Lam. (Berberidaceae) (Fig. 5C). Finally, Laguna del Huemul is part of the Altitude low scrub formation, and the Temperate Andean low scrub of *Discariachacaye* (G. Don) Tortosa (Rhamnaceae) and *Berberisempetrifolia* vegetal belt.

Our field observations in Laguna Añil (1724 and 1650 m elevation) indicate a total of 45 species associated with *Valerianapraecipitis*. Some of these species include: *Chiliotrichumdiffusum* (G. Forst.) Kuntze, *Senecio* spp. (Asteraceae), *Berberisempetrifolia*, *Maytenusdisticha* (Hook.f.) Urb. (Celestraceae), *Desfontainiafulgens* D. Don (Columelliaceae), *Empetrumrubrum* Vahl ex Willd., *Gaultheria* sp., *G.poeppigii* DC., *G.pumila* (L.f.) D.J. Middleton, *G.tenuifolia* (Phil.) Sleumer (Ericaceae), *Escalloniaalpina* Poepp. ex DC., *E.rubra* (Ruiz & Pav.) Pers., *Rayeniamalalcurensis* Menegoz & A.E. Villarroel (Escalloniaceae), *Luzula* sp. (Juncaceae), *Myrceugeniachrysocarpa* (O. Berg) Kausel, *Myrteolanummularia* (Poir.) O. Berg (Myrtaceae), *Nothofagusobliqua* (Mirb.) Oerst., *N.pumilio* (Nothofagaceae), *Codonorchislessonii* (Brongn.) Lindl. (Orchidaceae), *Ourisia* sp., *O.coccinea* (Cav.) (Plantaginaceae), *Chusqueamontana* Phil. (Poaceae), *Saxifragamagellanica* Poir. (Saxifragaceae), *Quinchamaliumchilense* Molina (Schoepfiaceae).

#### Conservation status.

*Valerianapraecipitis* is assessed here as Endangered (EN) under the IUCN categories following criteria B2ab(iii). Criterion B2 was selected because its area of occupancy is < 500 km^2^ (estimated at 20 km^2^). Criterion “a” was selected because it is known to exist in two locations (Fig. 1C, north and south of Ñuble river). Criterion “b(iii)” was selected because there is a projected decline in the area, extent, and quality of habitat. High Andean plants are very sensitive to global warming, given that migration is limited by the lack of connectivity between summits and its reduced areas on the top, additionally, the 2010–2017 mega-drought in Central Chile, resulted in a significant reduction in precipitation and snow cover – the most severe during the last 1000 years – together with an increase in temperatures for the last decade (Garreaud et al. 2017; Cordero et al. 2019). One of the climate change model scenarios projects an increase of at least 1 °C of the mean temperature for the next 30 years, plus a decrease in winter precipitations of about 30% at the end of the century (Bozkurt et al. 2017). *Valerianapraecipitis* extent of occurrence is ~555 km^2^ (Fig. 1B). The species is not present in any protected area in Chile, and it is not protected by law.

### Key to *Valeriana* species

Key to *Valeriana* species present in the Andes range of the Ñuble Region, according to the floristic inventories of Faúndez et al. (1994), Rondanelli et al. (2000), Rodríguez et al. (2008) and Villarroel (2019). Based on Kutschker (2008, 2011).

**Table:** 

1	Basal leaves strictly entire	**2**
–	Basal leaves strictly divided	**3**
–	Basal leaves entire and divided	**4**
2	Basal leaves membranaceous	***V.leucocarpa***
–	Basal leaves sub-fleshy or fleshy	**5**
5	Basal leaves fleshy	**6**
–	Basal leaves sub-fleshy	**7**
6	Basal leaves opaque green colour	***V.carnosa***
–	Basal leaves bright green colour	**8**
7	Basal leaves spatulate, ovate or suborbicular, with entire, sinuate or dentate margin; inflorescences paniculiform, densely contracted; fruits ellipsoid and glabrous	***V.chilensis***
–	Basal leaves ovate or elliptic, with a markedly sinuate or lobed margin; inflorescences paniculiform, lax; fruits tightly ellipsoid and densely hirsute	***V.hebecarpa***
8	Basal leaves spatulate-obovate, with entire margin; inflorescences spike-like and contracted	***V.macrorhiza***
–	Basal leaves spatulate, with entire to pausidentate margin; inflorescences glomeruliform and contracted	***V.fonckii***
3	Basal leaves fleshy	***V.praecipitis***
–	Basal leaves membranaceous	**9**
9	Stems hirsute, particularly at the nodes; basal leaf segments ovate to oblong; fruits ellipsoid	***V.valdiviana***
–	Stems with scarce pubescence; basal leaf segments ovate, oblong, lanceolate-elliptic; fruits ovoid and flat	***V.polemoniifolia***
4	Basal leaves membranaceous	**10**
–	Basal leaves sub-fleshy	**11**
10	Stems cylindrical and striated; basal leaf segments ovate-lanceolate; fruits ovoid or ellipsoid	***V.crispa***
–	Stems quadrangular with hairy winged edges; basal leaf segments oblong, ovate, lanceolate or suborbicular; fruits ellipsoid	***V.grandifolia***
11	Maximum plant height 80 cm; entire basal leaves oblong, ovate or elliptic; divided basal leaves pinnatilobate; fruits lageniform	***V.laxiflora***
–	Maximum plant height 40 cm; entire basal leaves ovate or elliptic; divided basal leaves pinnatisect; fruits ellipsoid	***V.obtusifolia***

## Discussion

Pollen evidence indicates that *Valeriana* is a Holarctic genus that might have arrived from the northern hemisphere, perhaps before the uplifting of the Andes during the Pleistocene (Kutschker and Morrone 2012). The authors found an endemism node area between latitudes 34° and 37° S, suggesting a diversification centre for *Valeriana* after a long period of isolation, caused by the Andes uplift. The same scenario might have occurred with other genera such as *Berberis* L., *Ribes* L., among others, indicating a complex centre of endemism of high conservation value (Moreira-Muñoz and Muñoz-Schick 2007). In the same way the genus *Valeriana* is key to understanding Andean biogeographic history (Kutschker and Morrone 2012), evolutionary processes that occurred in the area could have given rise to locally endemic species such as *V.praecipitis*, *Rayeniamalalcurensis* and *Violachillanensis* Phil. (Violaceae) (RBG Edinburgh 2021).

The discovery of *V.praecipitis* in southern South-America follows the recent discovery of *Rayeniamalalcurensis*, a newly described endemic genus and species found in the same area. As mentioned before, since the revision of Kutschker (2011), new locations and a new species were described. These discoveries suggest that both the region and the genus may reveal additional interesting botanic surprises. Although no genetic analysis was performed to determine its phylogenetic affinities, the comparison of morphological characteristics with its closest species shows notable differences in 11 out of 13 sets of characters (Table 1), in addition to a distance between their recorded distributions of approximately 410 km. The species is unmistakable, easy to recognize from other species in the area, especially in spring and summer due to its remarkable silvery-green basal leaves. However, it is difficult to find given the steepness of the sites where it occurs. Its extreme rarity and inaccessibility are undoubtedly the reasons why it has evaded discovery up until the present time.

In addition to consistent morphological differences, *V.praecipitis* stands out from other species of the genus due to its specific ecological habitat. Few other *Valeriana* species are able to grow abundantly in damp rock-cliffs at this high altitude, directly rooted in fissures or small soil pockets (e.g., *V.chilensis* Borsini in Chile or *V.ruizlealii* Borsini in Argentina). Rock-cliffs are challenging habitats with high erosion rates, limited soil depth and nutrients availability (Mathaux 2017; March-Salas et al. 2018). Mountain microclimatic conditions at high elevations are also severe due to high insolation and extreme low temperatures (Gale 1972). To adapt to these conditions, the species has a woody rhizome that can be more than 30 cm long and 5–20 mm in diameter, perhaps the largest of any *Valeriana* species. In addition, its deciduous leaves allow this species to avoid freezing temperatures and the presence of snow. These environmental conditions could have triggered the adaptation phenomenon mentioned above, and eventually speciation processes as suggested by Kutschker and Morrone (2012).

Little is known about rare and endangered species growing in the southern Andean cliff ecosystems, and many open questions remain. Future research needs to be done to identify morphological and physiological adaptations to grow under harsh soil and climatic conditions that could shed light on the future of Andean plant communities in the face of climate change. For instance, how new climatic scenarios could affect their area of occupancy? Are they able to migrate? Or more precisely, how extreme and persistent climatic conditions, like the ongoing mega-droughts, might affect the physiological performance of these species, perhaps pressing viable populations’ threshold to the edge. The recent finding of *V.praecipitis* and *Rayeniamalalcurensis*, in addition to other rare and endemic species, adds to the importance of monitoring and promoting the conservation of these species (Moreno-Gonzalez et al. 2019), especially those of Andean cliff ecosystems.

*V.praecipitis* is not present in any public protected area in Chile (Fig. 1B). In 2011, the Andean and pre-Andean sectors of the Ñuble Region and north of the Biobío Region were declared a UNESCO World Biosphere Reserve (San Martín 2014). All the known subpopulations of *V.praecipitis* are found within this Biosphere Reserve, except the one located in the surroundings of Laguna Añil. However, although they are internationally recognized, Biosphere Reserves protection is not guaranteed by Chilean legislation. Actually, all sites are in privately owned lands with an overall low level of anthropic change. Without any formal protection, the area is highly susceptible to be affected by the construction of hydroelectric dams, mining industry, unregulated animal farming and unsustainable tourism. The high biodiversity levels of this area (e.g., Villarroel 2019), including the presence of narrow range and threatened species such as *V.praecipitis* and *R.malalcurensis* and the high degree of threat of the Andean region, should give priority to the conservation of Andean territory of the Ñuble Region. Such conservation priority is ever more urgent in the light of future increasing trends of more frequent and severe fires (e.g., McWethy et al. 2021).

Another threat specific to cliff vegetation is the possible impact that rock-climbing could cause. In Chile (and worldwide) rock-climbing is of increasing interest, attracting more people to the mountains (Bogges et al. 2021), and climbers and hiker’s organizations are rightly asking for policy to change to allow for more open access to mountainous areas. However, with a greater number of people accessing mountain cliffs, the risk of negative human impacts would increase (Clark and Hessl 2015). So far, because of their remoteness and inaccessibility, Chilean cliff ecosystems have remained relatively unaltered by human impacts. The lack of access is not only due to a lack of trails, but also because many mountainous areas are privately owned. In the future, the major challenge will be to find the most effective strategy to conserve ecosystems, while maximizing the social benefits of access to mountains.

### Additional specimens examined

*Valerianaphilippiana*. **Argentina. Patagonia**: Neuquén Province, Cerro Colouhincul, Mar 1927, Comber E00143957 (E); Río Negro Province, Cerro López, Dic 1928, Cordini 1594532 (US). **Chile. Los Lagos**: Llanquihue Province, Parque Nacional Vicente Pérez Rosales – Volcán Osorno, 200 m elevation, 41°10'S, 72°30'W, Jan 1986, Gardner 107129 (CONC); Osorno Province, Parque Nacional Puyehue - Volcán Casablanca, 1500 m elevation, 40°47'S, 72°10'W, Jan 1988, Gardner & Knees E00023408 (E); Osorno Province, Mirador Puyehue, Feb 1971, Landrum 107764 (SGO).

## Supplementary Material

XML Treatment for Valeriana praecipitis
